# Prevalence and associations of adverse childhood experiences with anxiety and depressive symptoms in Indonesia

**DOI:** 10.3389/fpubh.2025.1714676

**Published:** 2025-12-10

**Authors:** Karen Arulsamy, Elmeida Effendy, Sarah Mardhiyah, Mustafa M. Amin, M. Surya Husada, Vita Camellia, Anne-Claire Stona, Eric Andrew Finkelstein

**Affiliations:** 1 Lien Centre for Palliative Care, Duke-NUS Medical School, Singapore, Singapore; 2 Department of Psychiatry, Universitas Sumatera Utara, Medan, Indonesia; 3 SingHealth-Duke Global Health Institute, Duke-NUS Medical School, Singapore, Singapore

**Keywords:** adverse childhood experiences, anxiety, depressive symptoms, mental health, abuse

## Abstract

**Background:**

Research in Southeast Asia—particularly Indonesia—on the link between adverse childhood experiences and adult mental health remains limited. The current study aims to examine the prevalence of ACEs in Indonesia, associations with anxiety and depressive symptoms among adults, and gender differences.

**Methods:**

We conducted an online cross-sectional survey of 872 Indonesians aged 21–64 years. ACEs were measured using the WHO ACE-IQ, and anxiety and depressive symptoms with the PHQ-2, GAD-2, and their composite PHQ-4. Associations between ACEs and symptoms were analyzed using weighted regression models adjusting for demographic variables.

**Results:**

ACEs were highly prevalent (79.5%), most commonly parental separation (50.9%), emotional neglect (39.4%), and sexual abuse (23.1%). Overall, 38.2% reported one ACE, 22.0% two, and 19.4% three or more. The experience of any ACE is associated with a 0.57-point increase (95% CI = 0.22–0.91) on the GAD-2 and a 0.59-point increase (95% CI = 0.30–0.88) on the PHQ-2. Any ACE is associated with a 10.0 percentage point (95% CI = 0.02, 0.18) increase in the likelihood of reporting at least mild symptoms of anxiety and a 6.6 percentage point (95% CI = 0.01–0.13) increase in the likelihood of reporting at least mild symptoms of depression. These associations are largest for individuals with 3 ACEs or more. Women face a disproportionate burden of ACEs, in terms of prevalence and cumulative exposure, and worse mental health symptoms.

**Conclusion:**

In summary, these findings highlight the importance of interventions that consider cumulative ACE exposure and adopt gender-sensitive approaches to reduce long-term mental health consequences.

## 1 Introduction

Adverse childhood experiences (ACEs) include exposure to domestic violence; abuse (physical, sexual, or emotional); neglect (physical or emotional); parental death, separation, or divorce; bullying; and growing up in a household affected by incarceration, mental illness, or substance misuse during the first 18 years of life (1). Globally, an estimated 40% of individuals have experienced at least one ACE, with higher prevalence rates documented in Asian countries (2–5). ACEs have large and persistent effects on health-risk behaviors and physical and mental outcomes, including premature mortality (6–8). Given their high prevalence and health effects, ACEs are increasingly recognized as a major public health concern (2, 6, 9).

Evidence from numerous international studies consistently shows that ACEs are associated with an increased risk of developing mental health conditions across the lifespan, including depression, anxiety, substance and alcohol use disorders, psychosis, and suicide attempts (6, 10–17). Early life trauma can cause neurobiological changes as well as cognitive and emotional dysfunction, contributing to poorer mental health (18–20). ACEs are also associated with structural and functional changes in the brain, driven by epigenetic modifications to gene expression, which can lead to systematic differences in how individuals experience and cope with stress (21–26). These can directly lead to an increased risk for mental health conditions and also indirectly through poor coping behaviors, such as smoking, excessive alcohol consumption, physical inactivity, and poor dietary habits (23, 27, 28). Outcomes are typically worse for individuals who have experienced a greater number of ACEs, with studies generally finding a dose-relationship relationship (1, 3, 12, 14, 29, 30).

Despite extensive evidence linking ACEs to adverse adult mental health outcomes, research in Southeast Asia—and Indonesia in particular—remains limited. Existing studies in Indonesia focus on children and adolescent samples and report prevalence rates ranging from 50–80 percent (31–33). Examining country-specific patterns in ACEs and their mental health effects is essential for developing trauma-informed policies, as both prevalence and outcomes may differ across social and cultural contexts (34, 35). For example, physical and emotional abuse may be more prevalent in some Asian populations due to strict parenting styles, where physical and verbal punishment remain common forms of discipline (36, 37). Given their prevalence and normalization, the impact of such experiences on mental health outcomes may be minimized or overlooked. Additionally, families may be reluctant to report abuse due to strong cultural emphasis on family values, reputation, and honor, which can contribute to a higher prevalence of more severe maltreatment (38, 39). In more patriarchal societies, gender differences in the experiences of ACEs and associated mental health impacts may also emerge (40).

To address these gaps, this study examines the prevalence of ACEs among adults in Indonesia, their associations with anxiety and depressive symptoms, and gender differences using data from a web panel survey and weighted analyses. We measure ACEs using the Adverse Childhood Experience International Questionnaire (ACE-IQ) and anxiety and depressive symptoms using the clinically validated Patient Health Questionnaire-4 (PHQ-4), which consists of the Patient Health Questionnaire-2 (PHQ-2) and Generalized Anxiety Disorder-2 (GAD-2) (41, 42). To address these gaps, this study examines the prevalence of ACEs among adults in Indonesia, their associations with anxiety and depressive symptoms, and gender differences using data from a web panel survey and weighted analyses. We measure ACEs using the Adverse Childhood Experience International Questionnaire (ACE-IQ) and anxiety and depressive symptoms using the clinically validated Patient Health Questionnaire-4 (PHQ-4), which consists of the Patient Health Questionnaire-2 (PHQ-2) and Generalized Anxiety Disorder-2 (GAD-2) (41, 42).

## 2 Methods

### 2.1 Participants

We conducted a cross-sectional web panel survey from April 23 to 28, 2025, aimed at Indonesian citizens aged 21 and above who are part of an online panel hosted by TGM Research. TGM Research is a market research company with a strong presence in Southeast Asia through its online panels. In Indonesia, the panel comprises over 270,000 active users—representing nearly 10% of the total population; 1,027 respondents were surveyed for this study. To recruit respondents, email invitations were sent to individuals who met the inclusion criteria (Indonesian citizens aged 21 and above). Consenting participants were directed to the survey which contained questions on ACEs, mental health symptoms, and demographics (gender, age, region, educational attainment, marital status, employment income, father's highest educational attainment, mother's highest educational attainment, and socioeconomic status during the first 18 years of life). The full survey took roughly 25 minutes to complete.

### 2.2 Measures

#### 2.2.1 Adverse childhood experiences (ACEs)

We measured ACEs based on the World Health Organization's Adverse Childhood Experience International Questionnaire – ACE IQ (43). The questions from ACE-IQ cover physical, sexual and emotional abuse and neglect by parents or caregivers; family dysfunction (violence against household members; living with household member who were substance abusers, mentally ill or suicidal, or imprisoned; having one or no parents, parental separation or divorce); bullying; and witnessing community violence and exposure to collective violence in the first 18 years of life. For the current study, we excluded questions on witnessing community violence as they are not that common in Indonesia (44). Response options to the ACE-IQ questions were either binary (yes or no) or categorical (e.g., many times, a few times, once, never; always, most of the time, sometimes, rarely, never). Scoring was done following the frequency version (43). Based on these questions, we first created binary variables to denote exposure to each of the 11 types of ACEs. Based on these variables, we derived two measures: an overall exposure measure to determine the experience of any ACE and a four-level categorical variable (0, 1, 2 and 3 or more experiences) to assess level of exposure. Our analyses incorporate (1) a variable capturing exposure to any ACE, (2) a variable capturing the number of ACEs (0, 1, 2, and 3 or more), and (3) multiple variables capturing each individual ACE, treated as separate independent variables.

#### 2.2.2 Mental health: depression and anxiety

Symptoms of depression and anxiety were measured using the Patient Health Questionnaire-4 (PHQ-4). The PHQ-4 consists of the 2-item Patient Health Questionnaire-2 (PHQ-2) and the Generalized Anxiety Disorder-2 (GAD-2). The PHQ-2 and GAD-2 have high sensitivity (83 percent and 88 percent respectively) and specificity (90 percent and 82 percent respectively) in detecting symptoms of anxiety and depression (42). Due to its brevity and ease of administration, the PHQ-4 is commonly used as a screener for symptoms of anxiety and depression including in clinical settings (45). Respondents were asked “Over the last two weeks, how often have you been bothered by the following problems?” and responded to four items (“Feeling down, depressed or hopeless”; “Little interest or pleasure in doing things”; “Feeling nervous, anxious or on edge; and “Not being able to stop or control worrying”). These items are scored on a scale with the response options “not at all”, “several days”, “more than half the days”, and “nearly every day”, which are scored as 0, 1, 2, and 3, respectively. This results in a score that ranges from 0 to 6 for each subscale – a score of ≥ 3 on either subscale is the validated threshold for detecting probable cases of anxiety and depression. The total score, obtained by summing the PHQ-2 and GAD-2, ranges up to 12. While it does not distinguish between anxiety and depression symptoms, it provides a useful measure of overall severity: scores of 3–5 indicate mild symptoms, 6–8 indicate moderate symptoms, and 9–12 indicate severe symptoms (42, 46). We assess five main anxiety and depression outcomes: continuous scores on the anxiety and depression subscales; the likelihood that respondents report at least mild symptoms of anxiety or depression (defined as a score of ≥3 on either subscale); and the likelihood that individuals fall into the mild (3–5), moderate (6–8), or severe categories (9–12) based on the total score. This measure is used specifically in the study to examine gender differences in symptom severity according to the number of ACEs reported.

#### 2.2.3 Covariates

We included gender, age, region, educational attainment, marital status, and employment income of the respondent as covariates, as these factors are commonly included in similar studies (5, 14, 47). These variables help account for potential demographic and socioeconomic influences on the relationships of interest. In addition, we adjusted for father's and mother's highest educational attainment and socioeconomic status during the first 18 years of life to account for early-life family background. These factors may confound the association between ACEs and adult mental health outcomes, as children from disadvantaged backgrounds may be more likely to experience ACEs and to develop subsequent psychological distress (48, 49).

### 2.3 Statistical analyses

We first present descriptive characteristics of the sample and overall and gender-specific prevalence rates. Prevalence rates are reported for experiencing any ACE, by the number of ACEs (0, 1, 2, or 3 or more), and for each type of ACE based on the frequency scoring method described in Section 2.2.1 (43). To examine the relationship between mental health symptoms (measured by scores on the anxiety and depression subscales, as well as the likelihood of reporting at least mild symptoms) and ACEs, we estimated ordinary least squares (OLS) regression models, regressing the PHQ-4 outcome variables on the three measures of ACEs described previously. For the binary outcome variables, this means we use a linear probability model where a change in the independent variable of interest corresponds to a given percentage point change in the outcome. We use an ordered probit model to assess gender differences in symptom severity across ACE exposure levels. Symptom severity (none, mild, moderate, severe) is regressed on the number of ACEs (0, 1, 2, ≥3) and covariates to estimate the probability of falling into each severity category by gender.

As some of the covariates may act as potential mediators, the model coefficients reflect the association between mental health and ACEs, both through and beyond the influence of these other variables. We accounted for parental educational attainment and early-life socioeconomic status, recognizing that greater economic insecurity in Indonesia may directly contribute to mental health symptoms. This represents a conservative approach, as these factors may also increase the likelihood of ACEs and, in turn, indirectly affect mental health. Multicollinearity among covariates was assessed using a matrix of pairwise correlations rather than calculating the variance inflation factor for each model. Following this approach, a correlation above 0.85 was considered indicative of multicollinearity. No multicollinearity was detected. The correlation matrix is provided in Supplementary material 1 (50). We used adjusted R-squared values and F-statistics (via adjusted Wald tests) as supporting evidence for model fit, given that our variables were selected based on prior studies (51–55). Adjusted R-squared values and F-statistics are presented in Supplementary material 2. Robust standard errors were used to account for heteroskedasticity.

All estimates were weighted using post-stratification weights for gender, age categories (5, 20–68), region (Java, Bali, Kalimantan, Kepulauan Maluku, Nusa Tenggara, Papua, Sulawesi, and Sumatra), and educational attainment (Tertiary qualification, less than tertiary qualification) based on the Indonesian population in 2024. Weighting parameters were obtained from the Department of Statistics Indonesia (BPS–Statistics Indonesia). While BPS publishes average employment income by subgroups, it does not provide the percentage of individuals within income categories. Therefore, we used educational attainment as a proxy for socioeconomic status in the weighting process. The population parameters used to create the post–stratification weights are provided in Supplementary material 2. All analyses were performed in Stata 15, with statistical significance denoted by ^*^, ^**^, and ^***^ corresponding to the 10%, 5%, and 1% levels, respectively.

We included two questions to identify respondents who were not paying attention to the survey: (1) ‘Suppose you were offered the chance to receive either IDR 100,000 today or IDR 80,000 in one year. Which would you choose?' and (2) ‘Suppose you were offered the chance to receive either IDR 100,000 today or IDR 60,000 in one year. Which would you choose?' Respondents who chose the smaller amount in either question were excluded. Of the 917 total respondents, 24 (2.6%) were excluded on this basis. Given the sensitivity of the ACEs questions, we provided a ‘Decline to answer' option. Respondents who selected the ‘Decline to answer' option for all questions for a particular ACE were excluded from the analysis, resulting in an additional 21 exclusions (2.3%). In total, 45 respondents (4.9%) were excluded based on these criteria resulting in 872 observations for analyses. There were no additional missing data, as all other questions were forced–choice. Prior to fielding the full survey, we conducted pilot tests with over 100 respondents to test and refine the survey. These responses were excluded from the analyses.

### 2.4 Ethics

Institutional Review Board (IRB) approval was obtained from the National University of Singapore (NUS–IRB−2024–790) and Universitas Sumatera Utara (112/KEPK/USU/2025) and informed consent was collected following IRB guidelines. The survey instrument and data can be shared upon reasonable request.

## 3 Results

### 3.1 Characteristics of the sample

Table 1 presents the characteristics of the sample prior to post–stratification weighting. The gender distribution closely mirrors that of the general population (50.5% male and 49.5% female). However, the sample is skewed toward younger individuals: 84.6% of respondents are under 50 years old, compared to 66.7% in the general population. Our sample is also more educated, with 60.4% holding a tertiary qualification, versus 10.2% in the general population. Among parents of respondents, close to 30% completed a tertiary qualification. In terms of income, the average monthly employment income reported by BPS–Statistics is IDR 3,040,719, indicating that our sample is more advantaged in this regard as well. Regional differences are smaller-−77.4% of respondents are from Java, compared to 66.2% in the general population. The proportion of respondents who are married is comparable to that of the general population. BPS–Statistics does not publish ethnic breakdowns.

**Table 1:** Characteristics of the sample (unweighted).

	**Sample percentage (%)**
**Gender**	
Female	49.54
Male	50.46
**Age**	
21–29	39.45
30–39	23.85
40–49	21.33
50–59	13.30
60–69	2.06
**Highest education completed (tertiary qualification)**	
Respondent	60.44
Respondent's mother	26.95
Respondent's father	29.13
**Household income**	
Less than IDR 1,000,000	8.72
IDR 1,000,000 to 1,999,999	9.63
IDR 2,000,000 to 2,999,999	11.35
IDR 3,000,000 to 3,999,999	12.61
IDR 4,000,000 to 4,999,999	11.01
IDR 5,000,000 to 5,999,999	12.61
More than 5,999,999	34.17
**Ethnicity**	
Javanese	51.03
Sundanese	19.38
Malay	7.00
Batak	5.39
Others	17.20
**Region**	
Java	77.41
**Marital status**	
Married	59.40
Separated	0.46
Widowed	2.06
Divorced	2.75
Single	35.32
Number of observations	872

Totals may not sum to 100 because of rounding.

### 3.2 Prevalence of ACEs

The following sections focus on weighted results, examining the prevalence and sociodemographic correlates of adverse childhood experiences, as well as their associations with anxiety and depressive symptoms. As shown in Table 2, the lifetime weighted prevalence of experiencing any ACE is 79.5%. In terms of number of ACEs, 38.2% of all respondents had experienced one ACE, 22% had experienced two ACEs while 19.4% had experienced three or more ACEs. The most common type of ACE experienced was parental separation, divorce or death (50.9%), emotional neglect (39.4%), and sexual abuse (23.1%). Sexual abuse includes questions on whether someone touched or fondled the respondent in a sexual way when the respondent did not want them to, or whether someone made the respondent touch them in a sexual way against the respondent's will. A large proportion of reported cases fall under these categories, rather than involving attempted or actual oral, anal, or vaginal intercourse. 21.4% reported that someone touched or fondled the respondent in a sexual way when the respondent did not want them to, 12.5% reported that someone made the respondent touch them in a sexual way against the respondent's will, 9.5% reported that someone attempted sexual intercourse with them, and 6.3% reported that someone had sexual intercourse with them against their will.

**Table 2:** Prevalence of adverse childhood experiences.

	***N* (Unweighted)**	**Weighted %**
Any ACE	697	79.47
**Number of adverse childhood experiences**		
0	193	20.53
1	302	38.15
2	215	21.96
3 or more	162	19.36
Emotional neglect	353	39.37
Physical neglect	101	12.52
Living with household members who were substance abusers	17	2.60
Living with household members who were mentally ill or suicidal	37	2.79
Witnessed domestic violence in the household	109	12.02
Living with household members who were imprisoned	15	1.96
Parental separation, divorce or death of a parent	379	50.85
Emotional abuse	45	4.59
Physical abuse	17	1.94
Sexual abuse	227	23.10
Bullying	35	1.80
Number of observations	872	

All results are weighted. Prevalence rates are reported for experiencing any ACE, by the number of ACEs (0, 1, 2, or 3 or more), and for each type of ACE based on the frequency scoring method described in Section 2.2.1.

### 3.3 Prevalence of ACEs by gender

Table 3 presents gender differences in the prevalence of overall, cumulative, and individual ACEs. At the aggregate level, women are 5.0 percentage points more likely to report any ACE, a difference that is marginally significant at the 10% level. Women are 13 percentage points more likely to report three or more ACEs (significant at the 1% level), while men are 9 percentage points more likely to report two more ACEs (significant at the 1% level). On average, women report 0.30 more ACEs than men—a statistically significant difference at the 1% level. Thus, women face a disproportionate burden of ACEs, both in terms of experiencing any ACE and cumulative exposure.

**Table 3:** Prevalence of adverse childhood experiences by gender.

	**Prevalence among males (%)**	**Prevalence among females (%)**	**Difference (standard error)**
Any ACE	77.06	81.97	0.05^*^ (0.27)
1 ACE	37.72	38.60	0.01 (0.03)
2 ACEs	26.48	17.25	−0.09^***^ (0.03)
3 or more ACEs	12.86	26.13	0.13^***^ (0.03)
Number of ACEs	1.39	1.69	0.30^***^ (0.09)
Emotional neglect	33.83	45.14	0.11^***^ (0.03)
Physical neglect	11.81	13.27	0.02 (0.02)
Living with household members who were substance abusers	3.74	1.42	−0.02^**^ (0.01)
Living with household members who were mentally ill or suicidal	4.35	1.16	−0.03^***^ (0.01)
Witnessed domestic violence in the household	5.76	18.54	0.13^***^ (0.02)
Living with household members who were imprisoned	3.05	0.82	−0.02^**^ (0.01)
Parental separation, divorce or death of a parent	54.77	46.76	−0.08^**^ (0.03)
Emotional abuse	1.18	8.14	0.07^***^ (0.01)
Physical abuse	0.95	2.98	0.02^**^ (0.01)
Sexual abuse	16.32	30.17	0.14^***^ (0.03)
Bullying	3.02	0.53	−0.03^***^ (0.01)
Number of observations	50.5	49.5	872

All results are weighted. Prevalence rates are reported for experiencing any ACE, by the number of ACEs (0, 1, 2, or 3 or more), and for each type of ACE based on the frequency scoring method described in Section 2.2.1. Standard errors are shown in parentheses. ^*^, ^**^, ^***^ indicates significance at the 10, 5 and 1 percent levels respectively.

More pronounced gender differences emerge when examining specific ACEs. Women are significantly more likely than men to experience sexual abuse, witness domestic violence in the household, and experience emotional neglect and abuse. These differences are substantial: women are 14.0 percentage points more likely to report sexual abuse, 13.0 percentage points more likely to have witnessed domestic violence, and 11 and 7.0 percentage points more likely to report emotional neglect and emotional abuse, respectively. In contrast, men are significantly more likely to report parental death or separation, living with household members who were substance abusers, mentally ill and/or suicidal, or incarcerated, as well as having experienced bullying. However, these differences are considerably smaller than those observed for men.

### 3.4 Associations between ACEs and symptoms of anxiety and depression

Tables 4, 5 present results from OLS models regressing each ACE variable on the anxiety score, depression score, and the presence of at least mild symptoms of anxiety and depressive symptoms based on a cut–off score of ≥ 3 on the PHQ−2 and GAD−2. Tables 4, 5 present results from OLS models regressing each ACE variable on the anxiety score, depression score, and the presence of at least mild symptoms of anxiety and depressive symptoms based on a cut–off score of ≥ 3 on the PHQ−2 and GAD−2 respectively. In Table 4, columns 2 and 4 present the PHQ−2 and GAD−2 scores, while columns 3 and 5 show the corresponding 95% confidence intervals for each outcome. The experience of any ACE is associated with 0.57–point increase (95% CI = 0.22, 0.91) on the GAD−2 and a 0.59–point increase (95% CI = 0.30, 0.88) on the PHQ−2. The magnitude of these associations is largest for individuals who have experienced 3 or more ACEs followed by 2 ACEs.

**Table 4:** Associations between ACEs and anxiety and depression scores.

	**Anxiety score**	**95% CI**	**Depression score**	**95% CI**
Any ACE	0.57^***^ (0.18)	(0.22,0.91)	0.59^***^ (0.15)	(0.30,0.88)
1 ACE	0.25 (0.20)	(−0.14,0.63)	0.30^*^ (0.16)	(−0.02,0.62)
2 ACEs	0.71^***^ (0.22)	(0.28,1.13)	0.82^***^ (0.21)	(0.41,1.22)
3 or more ACEs	1.00^***^ (0.24)	(0.53,1.46)	0.85^***^ (0.21)	(0.43,1.26)
Emotional neglect	0.48^***^ (0.17)	(0.16,0.81)	0.34^**^ (0.15)	(0.04,0.64)
Physical neglect	−0.36^*^ (0.21)	(−0.77,0.04)	−0.30^*^ (0.18)	(−0.65,0.05)
Living with household members who were substance abusers	0.57 (0.42)	(−0.24,1.39)	0.53 (0.52)	(−0.48,1.55)
Living with household members who were mentally ill or suicidal	1.22^**^ (0.51)	(0.21,2.23)	0.96^**^ (0.38)	(0.21,1.70)
Witnessed domestic violence in the household	0.66^***^ (0.24)	(0.14,1.11)	0.55^**^ (0.25)	(0.05,1.04)
Living with household members who were imprisoned	0.21 (0.39)	(−0.55,0.96)	0.17 (0.33)	(−0.47,0.81)
Parental separation, divorce or death of a parent	0.16 (0.16)	(−0.15,0.46)	0.26^*^ (0.15)	(−0.03,0.55)
Emotional abuse	0.94^**^ (0.38)	(0.01,0.41)	0.85^**^ (0.39)	(−0.09,0.26)
Physical abuse	0.69 (0.47)	(−0.23,1.60)	0.41 (0.32)	(−0.21,1.04)
Sexual abuse	0.56^***^ (0.20)	(0.17,0.94)	0.63^***^ (0.19)	(0.26,1.00)
Bullying	1.35^**^ (0.53)	(0.31,2.39)	0.63 (0.47)	(−0.30,1.51)

All results are weighted. The independent variables—experience of any ACE, number of ACEs (0, 1, 2, or ≥3), and each specific type of ACE—were constructed using the frequency scoring method described in Section 2.2.1. The dependent variables—anxiety and depression—were measured using the continuous GAD−2 and PHQ−2 scores, respectively. Standard errors are shown in parentheses. ^*^, ^**^, ^***^ indicates significance at the 10, 5 and 1% levels respectively.

**Table 5:** Associations between ACEs and likelihood of experiencing at least mild symptoms of anxiety and depression.

	**Presence of at least mild symptoms of anxiety**	**95% CI**	**Presence of at least mild symptoms of depression**	**95% CI**
Any ACE	0.10^***^ (0.04)	(0.02,0.18)	0.07^**^ (0.03)	(0.01,0.13)
1 ACE	0.06 (0.05)	(−0.04,0.15)	0.03 (0.04)	(−0.05,0.10)
2 ACEs	0.10^*^ (0.05)	(−0.02,0.20)	0.09^*^ (0.05)	(−0.01,0.19)
3 or more ACEs	0.19^***^ (0.06)	(0.06,0.31)	0.10^*^ (0.05)	(−0.01,0.207)
Emotional neglect	0.07 (0.04)	(−0.02,0.15)	0.02 (0.04)	(−0.05,0.10)
Physical neglect	−0.06 (0.06)	(−0.12,0.10)	−0.06 (0.04)	(−0.13,0.02)
Living with household members who were substance abusers	0.32^**^ (0.15)	(0.03,0.60)	0.23 (0.15)	(−0.08,0.53)
Living with household members who were mentally ill or suicidal	0.19 (0.15)	(−0.10,0.49)	0.28^**^ (0.13)	(0.02,0.54)
Witnessed domestic violence in the household	0.10 (0.07)	(−0.04,0.23)	0.08 (0.06)	(−0.04,0.19)
Living with household members who were imprisoned	−0.09 (0.10)	(−0.29,0.11)	−0.08 (0.29)	(−0.23,0.07)
Parental separation, divorce or death of a parent	0.01 (0.04)	(−0.07,0.09)	0.03 (0.04)	(−0.04,0.10)
Emotional abuse	0.20^**^ (0.10)	(0.01,0.41)	0.08 (0.09)	(−0.09,0.26)
Physical abuse	−0.03 (0.10)	(−0.23,0.18)	−0.02 (0.09)	(−0.19,0.15)
Sexual abuse	0.06 (0.05)	(−0.04,0.16)	0.10^*^ (0.06)	(−0.05,0.16)
Bullying	0.28^*^ (0.14)	(0.01,0.57)	0.12 (0.12)	(−0.12,0.36)

All results are weighted. The independent variables—experience of any ACE, number of ACEs (0, 1, 2, or ≥3), and each specific type of ACE—were constructed using the frequency scoring method described in Section 2.2.1. The dependent variables—the presence of at least mild symptoms of anxiety and depression—were defined using a cutoff score of ≥3 on the GAD−2 and PHQ−2 scales, respectively. Standard errors are shown in parentheses. ^*^, ^**^, ^***^ indicates significance at the 10, 5 and 1% levels respectively.

In Table 5, columns 2 and 4 present the likelihood of experiencing at least mild symptoms of anxiety and depression based on the cut–off score, while columns 3 and 5 show the corresponding 95% confidence intervals for each outcome. Any ACE is associated with a 10.0 percentage point increase (95% CI = 0.02, 0.18) in the likelihood of reporting at least mild symptoms of anxiety and a 7.0 percentage point increase (95% CI = 0.01, 0.13) in the likelihood of reporting at least mild symptoms of depression. Individuals who have experienced at least 3 ACEs are 19.0 percentage points (95% CI = 0.06, 0.31) more likely to report at least mild symptoms of anxiety and 10.0 percentage points (95% CI = −0.01, 0.207) more likely to report at least mild symptoms of depression. While the direction of the coefficients is positive for individuals who have experienced one ACE, these associations are largely not statistically significant. Multiple ACEs are positively correlated with both anxiety and depressive symptoms and individuals with such experiences are more likely to report anxiety symptoms than depression symptoms.

The associations between anxiety scores and bullying, having lived with household members who were mentally ill or suicidal, and emotional abuse are the largest in magnitude. Anxiety scores are also significantly positively correlated with having witnessed domestic violence in the household, sexual abuse, and emotional neglect. Having lived with household members who were substance abusers, bullying, and emotional abuse are significantly correlated with the likelihood of reporting at least mild symptoms of anxiety.

The associations between depression scores and having lived with household members who were mentally ill or suicidal, emotional abuse, and sexual abuse are also the largest in magnitude. Depression scores are also significantly positively correlated with having witnessed domestic violence in the household violence in the household, emotional neglect, and parental separation, divorce or death of a parent. In terms of the presence of clinical symptoms, only sexual abuse and having lived with household members who were mentally ill or suicidal are statistically significant.

The most common ACEs in the sample are parental separation, divorce or death of a parent, emotional neglect, and sexual abuse. The associations between ACEs and parental separation, divorce or death are largely not significant. While emotional neglect is associated with higher anxiety and depression scores, it is not associated with a higher likelihood of reporting at least mild symptoms of anxiety or depression. Sexual abuse is associated with higher anxiety and depression scores and a higher likelihood of reporting at least mild symptoms of depression significant at the 10% level.

### 3.5 Additional analysis: associations between ACEs and symptoms of anxiety and depression by gender

**Figure 1:**
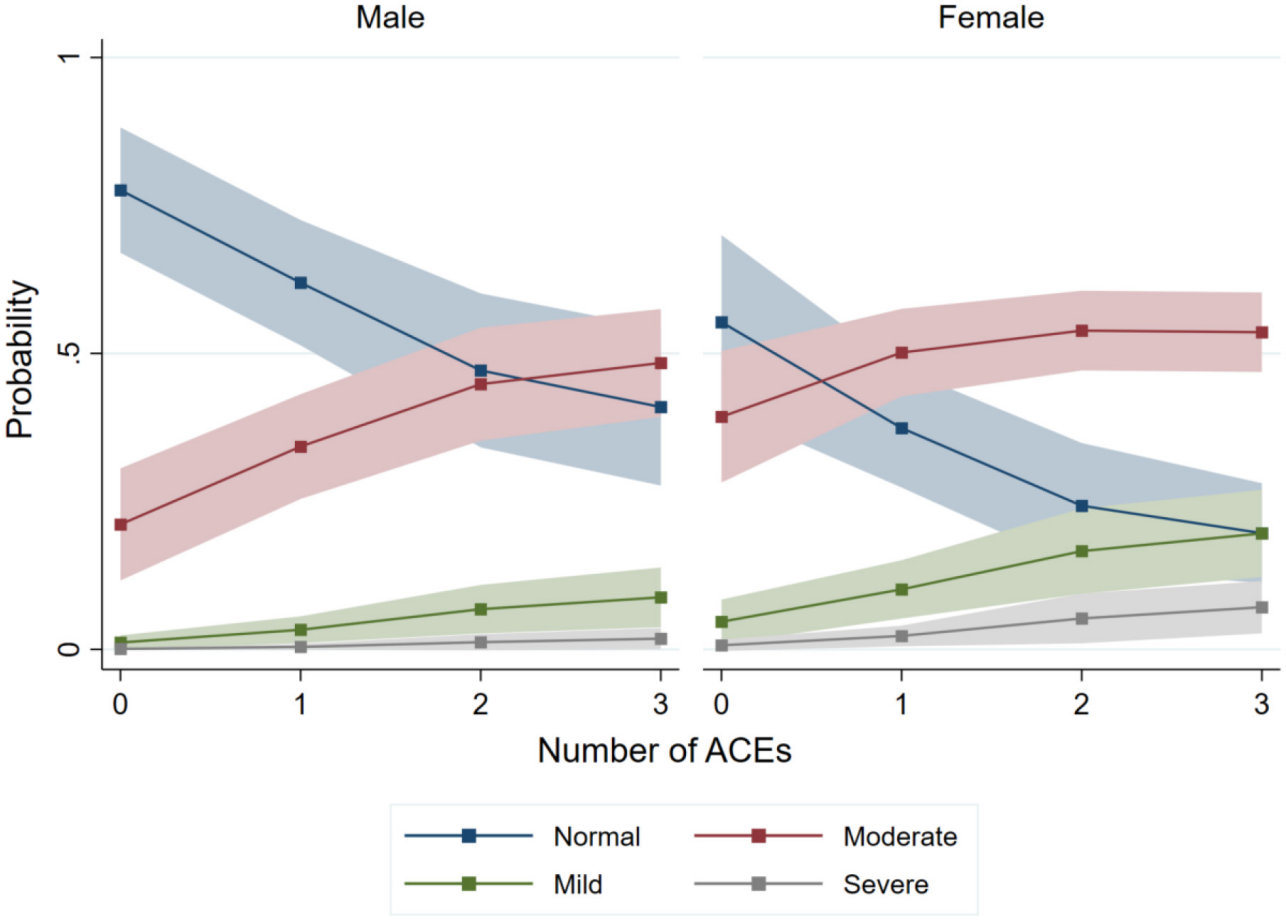
Gender differences in the probability of experiencing symptoms of anxiety and/or depression by number of ACEs. Results are weighed. The total score Severity is defined as follows: 0–2 indicates normal symptoms, 3–5 mild symptoms, 6–8 moderate symptoms, and 9–12 severe symptoms.

Given gender differences in both the prevalence and cumulative exposure to ACEs, we examined how mental health symptom severity varies by extent of ACE exposure for men and women. As shown in Figure 1, the probability of reporting greater levels of symptoms increase for all genders as the number of ACEs rises. However, the predicted probabilities of reporting mild, moderate, and severe symptoms—adjusted for covariates—are consistently higher for women with at least two ACEs compared to men. These findings suggest that women face not only a disproportionate burden of ACEs but also a higher likelihood of adverse mental health symptoms with cumulative exposure. For men, the probabilities of experiencing mild and severe symptoms remain broadly similar as the number of ACEs increases.

## 4 Discussion

In this study, we document a lifetime prevalence of any ACE of 79.5% The most common type of ACE experienced was parental separation, divorce or death (50.9%), emotional neglect (39.4%), and sexual abuse (23.1%). In terms of number of ACEs, 38.2 % of all respondents had experienced one ACE, 22% had experienced two ACEs while 19.4% had experienced three or more ACEs. The experience of any ACE is associated with a 0.57–point increase on the GAD−2 and a 0.59–point increase on the PHQ−2. Any ACE is associated with a 10.0 percentage point increase in the likelihood of reporting at least mild symptoms of anxiety and a 7.0 percentage point increase in the likelihood of reporting at least mild symptoms of depression. The magnitude of these associations is largest for individuals who have experienced 3 ACEs followed by 2 ACEs. While fairly common experiences, the associations between clinical levels of symptoms and parental separation, divorce, or death, emotional neglect and sexual abuse are attenuated. While less common, having lived with a household member who was mentally ill or suicidal, experienced emotional abuse, and having lived with household members who were substance abusers, and bullying have large and significant associations with reporting clinical symptoms. Notably, these associations are present even after accounting for parental educational attainment and early–life socioeconomic background. The consistency of our results across continuous PHQ−2 and GAD−2 scores, binary outcomes based on clinical cut–offs, and severity levels derived from the overall PHQ−4 score indicates that the associations between ACEs and symptoms of anxiety and depression are not unduly sensitive to the specific outcome measure used.

Importantly, we find a gender gradient in the experience of ACEs and associated mental health symptoms. Women are 5.0 percentage points more likely to report any ACE, a difference that is marginally significant at the 10% level. In terms of specific ACEs, women are significantly more likely than men to experience sexual abuse, witness domestic violence in the household, and experience emotional neglect and abuse. Women are also significantly more likely than men to report three or more ACEs. The predicted probabilities of reporting mild, moderate, and severe symptoms—adjusted for covariates—are consistently higher for women with at least two ACEs compared to men. Together, these findings indicate that women face a disproportionate burden of ACEs, in terms of prevalence and cumulative exposure, and a heightened likelihood of adverse mental health symptoms with cumulative exposure. These gender disparities are potentially shaped by cultural, structural, and societal factors in Indonesia. Prevailing gender norms and patriarchal structures may limit women's autonomy and increase their exposure to domestic and sexual violence. Social stigma surrounding disclosure of abuse may lead to underreporting and hinder access to support. Moreover, economic dependency, limited educational opportunities, and unequal power dynamics within households may also contribute to women's heightened vulnerability to cumulative exposure (56).

Our estimated prevalence exceeds both regional estimates and the global rate of 40% (2–5). Prior studies document an overall prevalence rate of 63.9% in Singapore, 75% in the Philippines, 76% in Vietnam, 38% in Thailand, and 74.6% in Hong Kong (3, 5, 57–59). The prevalence of parental death, separation, or divorce in our sample (50.9%) is notably higher than in Singapore (21.8%) and Hong Kong (23.8%) (5, 59). In contrast, the prevalence of emotional neglect (39.4%) is lower than Singapore (46.5%) but higher than Hong Kong (15.7%) (5, 59). Sexual abuse prevalence is considerably higher in our sample and warrants further research. For comparison, 3.9% reported sexual abuse in Singapore, 5.2% in the Philippines, 14.9% in Vietnam, 13.2% in Hong Kong, and 4.9% in Thailand (3, 5, 57–59). A large share of cases in our study involved unwanted touching or fondling or being forced to touch someone else in a sexual way. We also find a lower prevalence of emotional and physical abuse compared to other studies, more in line with findings from Singapore (3, 5, 57–59). Finally, the prevalence of witnessing domestic violence (12%) exceeds that reported in Singapore (8.2%) and Thailand (9.7%), is comparable to the Philippines (21.9%), and remains lower than in Hong Kong (30.5%) (3, 5, 57–59). These findings on witnessing domestic violence are consistent with prior evidence documenting elevated rates of physical, sexual, and psychological intimate partner violence in Indonesia (60).

While implemented using a low cost and expeditious approach, this study has limitations. The use of an online panel, rather than face–to–face interviews, resulted in a more socioeconomically advantaged sample—characterized by younger participants, those living in urban areas, and individuals with higher income and educational attainment. This may have contributed to differences in the experiences of ACEs. This could also partly explain why the reported occurrences of physical and emotional abuse are lower than in other studies. As such, the findings may not be generalizable to individuals without internet access, those from lower socioeconomic backgrounds, or those living in rural areas. That said, prior research suggests that interviewer–administered surveys may lead to greater underreporting of sensitive experiences compared with self–administered formats. Taken together, these factors could either inflate or deflate the observed ACE prevalence and its associations with mental health outcomes, making the net direction of any bias in our findings uncertain (61). ACEs were assessed via retrospective self–reporting, which may be subject to recall and reporting biases (62). Participants may inaccurately recall their ACE experiences, leading to over– or under–reporting. Psychological distress could further influence recall—for example, those experiencing distress may over–report ACEs, while those not in distress may under–report (63). Given the sensitive nature of ACEs, participants may be reluctant to share personal information and other factors such as personality traits may also influence how ACEs and mental health are self–reported (63–65). Without corresponding prospective data, it is challenging to gauge the direction of these biases.

Given the cross–sectional nature of the data, we are unable to establish causal relationships between ACEs and mental health outcomes and the relationships documented are associational. Many confounding factors beyond parental education and childhood socioeconomic status may influence the association between ACEs and symptoms of anxiety and depression, which we were not able to comprehensively account for in this study. While the PHQ−4 has relatively high sensitivity and specificity in detecting diagnosed cases, it is important to note that not everyone who screens positive for symptoms will receive a clinical diagnosis of anxiety or depression. Moreover, while it has been validated internationally and among diabetes patients in Indonesia, it has not been validated for use yet in the general Indonesian population (66). Furthermore, as our focus was on anxiety and depression symptoms, we did not collect information on other mental health conditions to assess comorbidity. Examining how ACEs relate to conditions such as substance use disorders, psychosis, and suicide attempts is an important area for future research. Given the gender disparities observed, it is important to understand underlying factors contributing to these differences to identify and design targeted interventions. We also did not collect information on respondents' membership in marginalized communities (e.g., religious minorities, people with disabilities, LGBTQ+ individuals) to examine whether the relationships between ACEs and mental health symptoms vary by these characteristics. Future studies should aim to corroborate these findings using alternative methodologies, including longitudinal designs and more representative samples drawn from national household surveys. Prospective longitudinal designs in particular may be helpful in clarifying causal relationships.

The high prevalence of ACEs in Indonesia underscores the urgent need for policies that prevent childhood adversity and mitigate its long–term mental health impacts. Given the disproportionate burden among women, gender–sensitive strategies are particularly important. Prevention and early intervention efforts can reduce the prevalence of mental health conditions and may benefit from a whole–systems approach, in which agencies involved in education, mental health, and social and family development are equipped to recognize ACEs and their effects. This is especially critical in light of the substantial economic costs of anxiety and depression documented in Indonesia (67, 68). Given the significant associations between the number of ACEs and symptom severity, routine screening for ACEs could help identify patients at greater risk for poor mental health outcomes and inform treatment plans (69, 70). Individuals with complex trauma may benefit more from trauma–focused counseling or psychotherapy rather than stand–alone pharmacological interventions. Trauma–focused interventions are shown to be more effective in treating mental health symptoms; however, only 0–22% of patients presenting with mental health symptoms are asked about ACEs (69, 71). There is also a need to build trauma–informed communities, bringing together mental health professionals, educators, child welfare services, and community providers to prevent ACEs and provide support to individuals with ACEs.

## 5 Conclusion

This study highlights the high prevalence of adverse childhood experiences in Indonesia and their significant associations with adult anxiety and depressive symptoms. The disproportionate burden borne by women emphasizes the need for gender–sensitive prevention and intervention strategies to reduce the long–term mental health impacts of childhood adversity.

## Data Availability

The raw data supporting the conclusions of this article will be made available by the authors, without undue reservation.
