# Supplementary material for: Prevalence and associations of adverse childhood experiences with anxiety and depressive symptoms in Indonesia
Source: Front Public Health. 2025 Dec 10;13:1714676. doi: 10.3389/fpubh.2025.1714676 (PMC12727598; doi:10.3389/fpubh.2025.1714676)
Supplement: Supplementary file 1 [file Data_sheet_1.docx]

Supplementary Materials

Supplementary Material 1

Table S1: Pairwise correlation matrix

|  | Sex | Age | Education | Marital Status | Employment Income | Mother’s education | Father’s education | Childhood socioeconomic status | Region | Ethnicity |
| --- | --- | --- | --- | --- | --- | --- | --- | --- | --- | --- |
| Sex | 1.00 |  |  |  |  |  |  |  |  |  |
| Age | -0.25 | 1.00 |  |  |  |  |  |  |  |  |
| Education | -0.14 | 0.45 | 1.00 |  |  |  |  |  |  |  |
| Marital Status | -0.00 | -0.42 | -0.06 | 1.00 |  |  |  |  |  |  |
| Employment Income | -0.13 | 0.22 | 0.12 | -0.24 | 1.00 |  |  |  |  |  |
| Mother’s education | -0.05 | -0.03 | 0.17 | 0.05 | 0.02 | 1.00 |  |  |  |  |
| Father’s education | -0.03 | -0.03 | 0.12 | 0.02 | 0.08 | 0.33 | 1.00 |  |  |  |
| Childhood socioeconomic status | 0.09 | -0.02 | 0.11 | 0.10 | 0.21 | 0.11 | 0.16 | 1.00 |  |  |
| Region | 0.02 | 0.15 | 0.18 | -0.09 | 0.05 | -0.01 | -0.02 | 0.08 | 1.00 |  |
| Ethnicity | -0.01 | 0.03 | -0.05 | 0.03 | 0.04 | -0.03 | -0.01 | -0.06 | -0.53 | 1.00 |

Supplementary Material 2: Adjusted R-Squared and F-Stat Results

Table S2: Associations between ACEs and anxiety and depression scores (Adjusted R-Squared and Likelihood Ratio Test (F-Stat))

|  | Anxiety scores | | Depression scores | |
| --- | --- | --- | --- | --- |
|  | Adj. R-Squared | F-Stat | Adj. R-Squared | F-Stat |
| Any ACE | 0.22 | 5.91 | 0.26 | 6.79 |
| 1 ACE | 0.25 | 6.13 | 0.29 | 5.88 |
| 2 ACEs | 0.25 | 6.13 | 0.29 | 5.88 |
| 3 or more ACEs | 0.25 | 6.13 | 0.29 | 5.88 |
| Emotional neglect | 0.22 | 5.95 | 0.25 | 5.38 |
| Physical neglect | 0.20 | 5.18 | 0.24 | 6.01 |
| Living with household members who were substance abusers | 0.20 | 5.61 | 0.24 | 5.06 |
| Living with household members who were mentally ill or suicidal | 0.21 | 5.08 | 0.25 | 5.81 |
| Witnessed domestic violence in the household | 0.21 | 4.95 | 0.25 | 5.39 |
| Living with household members who were imprisoned | 0.20 | 4.86 | 0.24 | 5.45 |
| Parental separation, divorce or death of a parent | 0.20 | 4.91 | 0.25 | 6.17 |
| Emotional abuse | 0.21 | 5.28 | 0.25 | 5.37 |
| Physical abuse | 0.69 | 4.97 | 0.41 | 5.44 |
| Sexual abuse | 0.21 | 5.32 | 0.24 | 7.19 |
| Bullying | 0.21 | 6.11 | 0.24 | 5.04 |

Notes: The independent variables—experience of any ACE, number of ACEs (0, 1, 2, or ≥3), and each specific type of ACE—were constructed using the frequency scoring method described in Section 2.2.1. The dependent variables—anxiety and depression—were measured using the continuous GAD-2 and PHQ-2 scores, respectively.

Table S3: Associations between ACEs and likelihood of experiencing at least mild symptoms of anxiety and depression (Adjusted R-Squared and Likelihood Ratio Test (F-Stat))

|  | Presence of at least mild symptoms of anxiety | | Presence of at least mild symptoms of depression | |
| --- | --- | --- | --- | --- |
|  | Adj. R-Squared | F-Stat | Adj. R-Squared | F-Stat |
| Any ACE | 0.15 | 3.11 | 0.18 | 3.14 |
| 1 ACE | 0.16 | 2.96 | 0.19 | 2.80 |
| 2 ACEs | 0.16 | 2.96 | 0.19 | 2.80 |
| 3 or more ACEs | 0.16 | 2.96 | 0.19 | 2.80 |
| Emotional neglect | 0.15 | 3.13 | 0.17 | 3.11 |
| Physical neglect | 0.14 | 3.33 | 0.18 | 3.23 |
| Living with household members who were substance abusers | 0.16 | 3.62 | 0.18 | 3.14 |
| Living with household members who were mentally ill or suicidal | 0.15 | 3.27 | 0.19 | 3.29 |
| Witnessed domestic violence in the household | 0.14 | 3.35 | 0.18 | 3.17 |
| Living with household members who were imprisoned | 0.14 | 3.32 | 0.17 | 3.15 |
| Parental separation, divorce or death of a parent | 0.14 | 3.31 | 0.17 | 3.17 |
| Emotional abuse | 0.15 | 3.60 | 0.18 | 3.18 |
| Physical abuse | 0.14 | 3.31 | 0.17 | 3.15 |
| Sexual abuse | 0.14 | 3.33 | 0.19 | 3.29 |
| Bullying | 0.15 | 3.37 | 0.18 | 3.06 |

Notes: The independent variables—experience of any ACE, number of ACEs (0, 1, 2, or ≥3), and each specific type of ACE—were constructed using the frequency scoring method described in Section 2.2.1. The dependent variables—the presence of at least mild symptoms of anxiety and depression—were defined using a cutoff score of ≥3 on the GAD-2 and PHQ-2 scales, respectively.

Table S4: Post-stratification inputs

| Characteristics | Proportions obtained from BPS-Indonesia (%) |
| --- | --- |
| Gender |  |
| Female | 50.5 |
| Age Categories |  |
| 20-29 | 23.2 |
| 30-39 | 22.7 |
| 40-49 | 20.7 |
| 50-59 | 16.6 |
| 60-69 | 10.8 |
| Educational attainment |  |
| At least tertiary qualification | 10.2 |
| Region |  |
| Java | 66.2 |
